# Supplementary material for: Combined Approach: FFQ, DII, Anthropometric, Biochemical and DNA Damage Parameters in Obese with BMI ≥ 35 kg m−2
Source: Nutrients. 2023 Feb 10;15(4):899. doi: 10.3390/nu15040899 (PMC9958661; doi:10.3390/nu15040899)
Supplement: Supplementary file 1 [file nutrients-15-00899-s001.zip › nutrients-2173831-supplementary.pdf]

Table S1. Individual demographic details

Code	G	age	S	education	exposure	Physical activity (walking)	disease	therapy	tumors
**1**	f	60	No	Middle	No	No	no	no	No
**2**	m	55	No	Middle	Pesticides	No	Hypertension, 10y, hyperlipoproteinemia	Diuver, Loniten, Nebivolol, Lercanil, Moxaviv, Perineva, Cholib	No
**3**	f	64	No	Middle	No	No	Diabetes,20y and hypertension 20y, hyperlipoproteinemia	Belformin, Abasaglar, Ramed, Lacipil, Moxaviv, Cholib	Thyroid tumor 10 y ago
**4**	m	48	No	High	No	to workplace	Asthma, 20y, hypertension 20y, hyperlipoproteinemia	Diuver 5 mg 1 tbl, Co-Articel 10/2.5 mg 1 tbl	No
**5**	m	66	No	Middle	No	No	Diabetes 17y, hypertension, hyperlipoproteinemia	Triplixam, Synjardy, Trulicity, Choli, Tomid	No
**6**	f	65	No	Low	No	No	Asthma, hypertension, hyperlipoproteinemia	Edemid F, Epri,, Cozaar	No
**7**	m	42	No	Middle	Pesticides	No	Hypertension, 10y	Tomid, Perineva, Moxaviv, Nebyol	No
**8**	m	66	No	Middle	No	No	hypothyroidism, 12 y ago, hypertension, hyperlipoproteinemia	Articel, Indapamid, Physiotens, Nibel	Thyroid carcinoma, 12 y ago
**9**	m	51	No	Middle	No	No	No	No	No
**10**	f	61	No	High	Pesticides	No	Diabetes, 14y, and hypertension, 14y, and anemia, 2y	Fursemide, Diglical Actos, Bydureon, Siofor, Articel, Atorvox, Byol	colon carcinoma 14 y, nose tumor 4y ago
**11**	f	52	Yes	Middle	No	to workplace	Hypertension, diabetes 10y, hyperlipoproteinemia	Synjardy, Siofor, Norprexanil	No
**12**	m	55	No	Middle	Pesticides	No	Chronic sinusitis, psoriasis vulgaris, arthritis psoriatica, diabetes, 4y, hypertension, hyperlipoproteinemia	PIRAMI, Lacidipin, Nebilet plus, Cholib, Siofor	No
**13**	f	56	No	Low	No	No	Asthma, diabetes 6y, hypertension, hyperlipoproteinemia	Fursemide, Trajenta	Uterus myoma
**14**	f	67	No	Middle	No	No	Hypertension, 5y, hyperlipoproteinemia	Perineva	Ovary 12y and Breast 7y ago

**15**	m	57	No	Middle	No	No	Diabetes II, 10y¸ hypertension, hyperlipoproteinemia	Co-Articel, Siofor	No
**16**	f	46	No	Middle	No	No	Diabetes 5y¸ hypertension, hyperlipoproteinemia	Triplixam, Siofor	No
**17**	m	50	No	Middle	Pesticides	No	Diabetes 13y¸ hypertension, hyperlipoproteinemia	Prylar, diuver, Novomix, Synjardy Siofor, Rosix	No
**18**	m	68	No	Middle	No	to workplace	Diabetes, 20 y, hypertension, hyperlipoproteinemia	Edemid F, Byol, Atorvox, Co-perineva, Novomix, Novorapid, Trulicity, Forxiga	No
**19**	m	59	No	Middle	No	No	Diabetes, 15 y, hypertension, hyperlipoproteinemia	Diglical, Trulicity, Xigduo, Actos, Siofor, Coupet, Co-Perineva	No
**20**	f	61	No	High	No	No	Diabetes 15y, hypertension 15y, and arthrosis	Fursemide, Atorvox, Diglical, Trulicity, Siofor, Articel-Am, Byol	No
**21**	m	41	Yes	Middle	No	No	Hyperlipoproteinemia new diagnosis	No	Colon 14y and nose carcinoma 4y ago
**22**	f	64	No	Middle	No	to workplace	Diabetes,20y and hypertension 20y, hyperlipoproteinemia	Trulicity, Abasaglar, metformin, Furosemide, Co-Dalneva, Moxaviv, Cholib	fibroadenoma
**23**	f	64	No	Middle	No	to workplace	Diabetes 20 y, hypertension 20 y, hyperlipoproteinemia	Furosemide, moxonidine, perindopril, indapimide/amlodipine, fenofibrate/simvastatin, metformin, glargine, semaglutide, trimetazidine, fluoxetine, oxazepam, levothyroxine, pantoprazole, cholecalciferol, beclometasone/formoterol, montelukastum, lactulose	thyroid tumor 10 y ago
**24**	m	43	No	Middle	No	No	Diabetes, hypertension 10 y, gout	Torasemide, nebivolol, allopurinol	No
**25**	f	58	No	Middle	No	No	Hypertension 7 y, fibrilacija atrija, Diabetes, hyperlipoproteinemia, gout	Furosemide, valsartan/hydrochlorothiazide, rosuvastatin, metformin, bisoprolol, amiodarone, dabigatran, clopidrogel	No

**26**	f	60	No	Middle	No	Everyday	Diabetes 4y, hypertension , hyperlipoproteinemia, hypothyroidism	perindopril, indapimide/amlodipine, atorvastatin, metformin, semaglutide, bisoprolol, levothyroxine, febuxostat	No
**27**	f	29	No	Middle	No	3-4 times per week	No	zoloft	No
**28**	f	41	No	Middle	No	No	No	Seroxat, Co-perineva	No
**29**	f	39	No	Middle	No	3-4 times per week	No	Knavom	No
**30**	f	44	No	High	Pesticides	3-4 times per week	Hypertension 8 y	Article, Claritine	No
**31**	f	46	No	High	No	No	No	Physiotens, Norvasc,Coupet	No
**32**	f	46	No	High	No	3-4 times per week	No	No	No
**33**	m	26	No	Low	No	No	No	No	No
**34**	f	65	No	High	No	3-4 times per week	Hypertension 10 y	Piramil, Tonocardin, ketonal, ibruprofen	No
**35**	f	64	No	High	No	No	No	Osan Plus, Byol Cor	No
**36**	f	49	No	High	Pesticides	No	Endometriosis 25 y	No	No
**37**	f	36	No	High	No	No	No	ketonal, andol	No
**38**	f	41	No	High	No	Everyday	No	No	No
**39**	m	51	No	Middle	Wood or metal varnish	Light hiking once per week	No	Piramil, Nibel, Amlopin, ketonal, klavocin	No
**40**	f	36	Yes	Middle	No	3-4 times per week	Hashimoto's thyroiditis	ibruprofen	No

**41**	f	63	No	Low	No	3-4 times per week	No	Physiotens, Co-Articel, Nebilet, alopunrinol	No
**42**	f	35	No	Middle	No	Everyday	No	Ormidol, Nalgesin, eutirox	No
**43**	f	30	No	High	No	No	Hypothyroidism 6 y	letrox	No
**44**	f	38	No	Middle	No	No	Hypertension, anxiety disorders	Cipralex, Prilen	No
**45**	f	61	No	Low	No	No	No	No	No
**46**	f	48	No	High	No	No	Hypothyroidism	Bisobel, Article, rupurut	Dermoid cyst ovary
**47**	f	58	No	High	No	No	Pulmonary Sarcoidosis 2 y, hypothyroidism 20 y, Hypertension 7 y	Co-Article, eutirox	No
**48**	m	61	No	High	No	No	No	No	No
**49**	m	60	No	High	No	Everyday walking	Hypertension 10 y	Norprexanil	No
**50**	f	49	No	Middle	No	No	Hypertension	Piramil, Tricor	No
**51**	f	40	No	High	No	No	Asthma	Foster, Singulair, Xyzal, ventolin- astma	No
**52**	f	46	No	Middle	Wood or metal varnish	No	anxiety disorders, hypertension, polycystic ovary syndrome	Seroxat, helex, Ormidol	No
**53**	f	27	No	High	No	Everyday	No	No	No

G-gender, f-female, m-male, S-smoking, y-years

Table S2. Average consumption of food groups from FFQ questionnaire in last year of 130 commonly and less commonly used foods in 12 categories for entire group (n=53)

**FOODS AND AMOUNTS**		**monthly**		**weekly**			**daily**			
		**Never, <1**	**1-3**	**Once**	**2-4**	**5-6**	**Once**	**2-3**	**4-5**	**6 +**
**MEAT AND FISH**	Beef: roast, steak, mince (120g.), stew (330g.) or casserole	11	15	10	14	1	1	1	0	0
**(medium serving)**	Beefburgers	40	9	4	0	0	0	0	0	0
	Pork: roast, chops, stew or slices [chop, (120g.) goulash (330g.)]	5	16	17	11	3	1	0	0	0
	Lamb: roast, chops or stew [chop (120g.), goulash (330g.)]	43	8	2	0	0	0	0	0	0
	Chicken or other poultry eg. Turkey (130 g.)	5	6	34	6	0	1	1	0	0
	Bacon (40 g.)	9	15	18	10	0	1	0	0	0
	Ham (40 g.)	12	16	13	6	5	1	0	0	0
	Corned beef, Spam, luncheon meats [canned/luncheon meat (40 g.)]	38	13	1	1	0	0	0	0	0
	Sausages (45 g.)	9	25	13	5	0	0	1	0	0
	Savoury pies, eg. Meat/pork pie, pasties, steak & kidney pie, sausage rolls [leafy meat-pie 160 g.)]	28	18	5	2	0	0	0	0	0
	Liver, liver paté, liver sausage (40 g.)	21	19	8	4	0	1	0	0	0
	Fried fish in batter, as in fish and chips	25	17	10	1	0	0	0	0	0
	Fish fingers, fish cakes	29	14	10	0	0	0	0	0	0
	Other white fish, fresh or frozen, eg. cod, haddock, plaice, sole, halibut	35	11	7	0	0	0	0	0	0
	Oily fish, fresh or canned, eg. mackerel, kippers, tuna, salmon, sardines, herring	16	24	13	0	0	0	0	0	0
	Shellfish, eg. crab, prawns, mussels	39	11	2	1	0	0	0	0	0
	Fish roe, taramasalata	52	0	1	0	0	0	0	0	0
**BREAD AND**	White bread and rolls	13	3	5	7	2	8	12	3	0
**SAVOURY BISCUITS**	Brown bread and rolls	14	13	5	7	2	4	8	0	0
**(one slice or biscuit)**	Wholemeal bread and rolls	19	6	6	7	2	5	7	1	0
	Cream crackers, cheese biscuits	27	13	7	4	1	0	0	1	0
	Crispbread, crackers	35	10	5	2	0	0	0	1	0
**CEREALS**	Porridge, Readybrek [palenta]	16	23	10	4	0	0	0	0	0

**(one bowl)**	Breakfast cereal such as cornflakes, muesli etc.	34	12	2	4	1	0	0	0	0
**POTATOES, RICE AND PASTA (medium serving)**	Boiled, mashed, instant or jacket potatoes (180 g.)	4	8	11	22	5	3	0	0	0
	Chips, Pommes frites (100 g.)	20	10	13	5	3	1	1	0	0
	Roast potatoes (180 g.)	6	14	28	3	2	0	0	0	0
	Potato salad (120 g.)	15	16	19	3	0	0	0	0	0
	White rice (150 g.)	5	19	21	7	1	0	0	0	0
	Brown rice (150 g.)	47	3	2	1	0	0	0	0	0
	White or green pasta, eg. spaghetti, macaroni, noodles) (230 g.)	9	12	20	11	1	0	0	0	0
	Wholemeal pasta [whole-grain pasta (230 g.)]	45	4	1	3	0	0	0	0	0
	Lasagne, moussakaa (400 g.)	27	21	5	0	0	0	0	0	0
	Pizza (240 g.)	25	16	11	1	0	0	0	0	0
**DAIRY PRODUCTS AND FATS**	Single or sour cream (tablespoon)	12	16	12	10	0	2	1	0	0
	Low fat yogurt, fromage frais (125g carton)	21	5	9	10	6	1	1	0	0
	Full fat or Greek yogurt (125g carton)	32	6	8	4	2	1	0	0	0
	Dairy desserts (125g carton)	30	10	8	3	1	1	0	0	0
	Cheese, eg. Cheddar, Brie, Edam (medium serving) [tilzit, gouda (40g.)]	7	13	8	11	7	5	2	0	0
	Cottage cheese, low fat soft cheese (medium serving) [(40g.)]	10	11	15	11	4	1	1	0	0
	Eggs as boiled, fried, scrambled, etc. (one)	3	12	11	20	4	3	0	0	0
	Quiche (medium serving) (120g.)	48	5	0	0	0	0	0	0	0
	Low calorie, low fat salad cream (tablespoon) (15g.)	51	0	1	0	1	0	0	0	0
	Salad cream, mayonnaise (tablespoon) (30g.)	48	3	2	0	0	0	0	0	0
	French dressing (tablespoon) (15g.)	51	1	1	0	0	0	0	0	0
	Other salad dressing (tablespoon) (15g.)	43	2	1	4	1	2	0	0	0
**The following on bread or vegetables**	Butter (teaspoon) (15g.)	23	16	8	4	1	1	0	0	0
	Block or hard margarine, eg. Stork, Krona (teaspoon) (15g.)	29	11	8	4	1	0	0	0	0
	Polyunsaturated margarine, eg. Flora, Omegol, sunflower, soya spreads (teaspoon) (15g.)	42	4	4	2	1	0	0	0	0

	Soft margarines, olive oil based and dairy spreads (teaspoon) [dairy spreads (15g.)]	41	5	3	2	1	1	0	0	0
	Low fat spreads (less than 60% fat) (teaspoon) (15g.)	33	9	4	5	0	2	0	0	0
	Very low fat spread (less than 30% fat) (teaspoon) (15g.)	34	11	1	4	1	2	0	0	0
**SWEETS AND SNACKS**	Sweet biscuits, chocolate, eg. digestive (one)	17	12	8	4	6	3	3	0	0
**(medium serving)**	Sweet biscuits, plain, eg. Nice, ginger [petit beurre] (one)	22	17	6	4	1	1	2	0	0
**[slice or 1 piece]**	Cakes eg. fruit, sponge, home baked	31	15	6	0	0	0	1	0	0
	Cakes eg. fruit, sponge, ready made	42	7	3	0	0	0	1	0	0
	Buns, pastries eg. scones, flapjacks, home baked	15	23	9	4	1	0	1	0	0
	Buns, pastries eg. croissants, doughnuts, ready made	22	13	11	4	0	1	2	0	0
	Fruit pies, tarts, crumbles, home baked	34	14	4	0	0	0	1	0	0
	Fruit pies, tarts, crumbles, ready made	40	9	2	1	0	0	1	0	0
	Milk puddings, eg. rice, custard, trifle (200g.)	36	8	6	2	0	0	1	0	0
	Ice cream, choc ices (60 g)	19	15	11	5	0	2	1	0	0
	Chocolates, single or squares (50 g)	12	12	9	7	2	4	6	0	1
	Chocolate snack bars eg. Mars, Crunchie, Mars, Twix…	31	6	5	4	1	3	2	0	0
	Sweets, toffees, mints (8 g)	32	6	8	2	1	1	3	0	0
	Sugar added to tea, coffee, cereal (teaspoon)	31	4	1	3	1	4	5	3	1
	Crisps or other packet snacks [chips, flips](40 g)	17	19	6	7	2	1	1	0	0
	Peanuts or other nuts (20 g)	12	22	2	8	2	3	3	1	0
**SOUPS, SAUCES, AND**	Vegetable soups (bowl) (220g.)	10	19	15	8	0	1	0	0	0
**SPREADS**	Meat soups (bowl) (220g.)	5	12	18	13	2	3	0	0	0
	Sauces, eg. white sauce, cheese sauce, gravy (tablespoon) (60g.)]	25	16	9	3	0	0	0	0	0
	Tomato ketchup (tablespoon) (30g.)	30	6	11	6	0	0	0	0	0
	Pickles, chutney [*Ajvar, pinđur* (15g.)]	17	14	11	8	1	1	1	0	0
	Jam, marmalade, honey (teaspoon) (15g.)	22	11	11	5	0	3	1	0	0
	Peanut butter (teaspoon) (15g.)	44	4	2	1	1	1	0	0	0

	Tea (cup)	14	3	5	10	5	10	5	1	0
**DRINKS**	Coffee, instant or ground (cup)	6	2	0	3	8	9	24	1	0
	Coffee, decaffeinated (cup)	47	1	0	2	0	3	0	0	0
	Cocoa, hot chocolate (cup)	41	6	0	3	1	1	1	0	0
	Wine (glass) (125 ml)	29	8	6	5	2	1	1	1	0
	Beer, lager or cider (half pint) (250 ml)	39	7	5	1	0	0	1	0	0
	Port, sherry, vermouth, liqueurs (glass)	43	6	4	0	0	0	0	0	0
	Spirits, eg. gin, brandy, whisky, vodka (single)	34	12	5	0	0	2	0	0	0
	Low calorie or diet fizzy soft drinks (glass)	35	6	2	4	0	3	2	1	0
	Fizzy soft drinks, eg. Coca cola, lemonade (glass)	23	8	10	4	3	1	3	1	0
	Pure fruit juice (100%) (glass)	26	7	11	5	2	2	0	0	0
	Fruit juice (100%) eg. orange, apple juice (glass)	33	8	5	3	1	2	1	0	0
	Fruit squash or cordial (glass)	31	5	5	6	1	2	2	1	0
**FRUIT**	Apples (1 fruit)	6	12	8	12	1	9	5	0	0
For seasonal fruits	Pears (1 fruit)	20	16	9	5	1	1	1	0	0
marked *, please estimate	Oranges, satsumas, mandarins (1 fruit)	10	6	14	12	4	6	1	0	0
your average use when	Grapefruit (half)	41	9	2	0	1	0	0	0	0
the fruit is in season	Bananas (1 fruit)	3	11	14	16	2	7	0	0	0
	Grapes (medium serving) (100 g)	17	19	11	4	0	1	0	1	0
	Melon (1 slice)	34	13	3	2	1	0	0	0	0
	* Peaches, plums, apricots (1 fruit)	19	13	11	7	1	2	0	0	0
	* Strawberries, raspberries, kiwi fruit (medium serving) (80 g)	21	9	14	7	0	1	1	0	0
	Tinned fruit (medium serving) [(100 g)]	42	7	4	0	0	0	0	0	0
	Dried fruit, eg. raisins, prunes (medium serving) (30g)	30	17	2	3	1	0	0	0	0
**VEGETABLES**	Carrots (60g.)	7	5	6	20	10	4	0	0	1
	Spinach (90g.)	14	21	15	2	1	0	0	0	0

Fresh, frozen or tinned (medium serving)	Broccoli, spring greens, kale (90g.)	16	14	18	5	0	0	0	0	0
	Brussels sprouts (90g.)	23	16	11	3	0	0	0	0	0
	Cabbage (90g.)	5	13	23	11	1	0	0	0	0
	Peas (65g.)	8	23	19	2	1	0	0	0	0
	Green beans, broad beans, runner beans (90g.)	13	18	17	5	0	0	0	0	0
	Marrow, courgettes (224g.)	14	15	16	8	0	0	0	0	0
	Cauliflower (90g.)	13	19	16	5	0	0	0	0	0
	Parsnips, turnips, swedes (70g.)	35	6	7	3	2	0	0	0	0
	Leeks (160g.)	14	19	15	5	0	0	0	0	0
	Onions (90g.)	2	3	11	14	7	15	0	0	1
	Garlic (10g.)	6	5	10	17	1	13	0	0	1
	Mushrooms (44g.)	12	27	11	3	0	0	0	0	0
	Sweet peppers (160g.)	9	17	17	9	0	1	0	0	0
	Beansprouts (60g.)	46	1	2	3	1	0	0	0	0
	Green salad, lettuce, cucumber, celery (30g.)	5	5	5	21	7	9	1	0	0
	Watercress (20g.)	43	2	1	5	1	1	0	0	0
	Tomatoes (85g.)	3	5	13	21	5	4	2	0	0
	Sweetcorn (60g.)	27	12	9	5	0	0	0	0	0
	Beetroot (35g.)	10	22	9	10	2	0	0	0	0
	Coleslaw (45g.)	4	11	15	18	3	1	1	0	0
	Avocado (145g.)	47	4	1	1	0	0	0	0	0
	Baked beans (135g.)	31	18	4	0	0	0	0	0	0
	Dried lentils, beans, peas (30g.)	28	14	10	1	0	0	0	0	0
	Tofu , soya meat, TVP, Vegeburger (60g.)	52	1	0	0	0	0	0	0	0

**MILK**	Full cream/whole (3,5 % -4,0 % m.m.)	5	Semi-skimmed (1,5%-2,8% m.m.)	29
	Skimmed (do 0,5 % m.m.)	2	Other	3

Dried	0	Soya	1

Table S3 Biochemical parameters on the group level (n=53) with Spearman correlation with all significant correlations found

**Variables**, *referent values*	**Mean**	**Median**	**Min**	**Max**	**SD**	**SE**
*Erythrocytes, 3.86-5.08F, 4.34-5.72M *10e12/L*	4.85	4.82	3.86	**5.81**	0.41	0.06
Hemoglobin, *119-157F, 138-175M g/L*	139.04	138.00	107.00	165.00	13.65	1.88
Hematocrit, *0.356-0.47F, 0.414-0.53M L/L*	0.41	0.41	0.33	0.48	0.03	0.00
MCV, *83-97.2FM fl*	84.63	85.30	62.80	94.30	6.14	0.84
**Spearman correlation:** ***hemoglobin(0.35),*** *mono% (0.29),* ***erythrocytes(-0.46), RDW(-0.38),*** *neutrophils(-0.29),* ***insulin(-0.56), HOMA-IR(-0.51)***						
RDW %, *9-15FM*	13.91	13.50	12.40	**19.70**	1.28	0.18
**Spearman correlation:** *inorganic phosphates(0.30),* ***chlorides(0.42****), HOMA-IR(0.31), BFM(0.28), BMI(0.32),* ***MCV(-0.38)***						
Platelets, *158-424FM *10e9/L*	272.30	249.00	167.00	**563.00**	86.76	11.92
**Spearman correlation:** ***leukocytes(0.61), neutrophils(0.58),*** ***neutrophils%(0.36), monocytes(0.41), hsCRP(0.37),*** *TC(0.30), lymphocytes(-0.29),* ***monocytes%(-0.36), urates(-0.39), inorganic phosphates(-0.36), chlorides(-0.39),*** *hs troponin(-0.28), SMM(-0.29), BMR(-0.32*)						
*Leukocytes, 3.4-9.7FM *10e9/L*	8.20	7.70	5.04	**14.26**	2.12	0.29
**Spearman correlation:** ***platelets(0.61), neutrophils(0.91), %neutro(0.44), lymphocytes(0.52), monocytes(0.71), hsCRP(0.38),*** *W-H-ratio(0.32), basophils%(-0.30), lymphocytes%(-0.33),* ***mono%(-0.43)***						
Basophils, 0-0.06FM, *10e9/L	0.03	0.03	0.01	0.09	0.02	0.00
**Spearman correlation:** ***hs troponin(0.37),****fT3(-0.28)*						
Basophils %, 0-1FM	0.44	0.40	0.10	**1.30**	0.24	0.03
**Spearman correlation:** ***hs troponin(0.36),*** *leukocytes(-0.30),* ***neutrophils(-0.36),*** *neutrophils%(-0.33), TC(-0.29), LDL-C(-0.30)*						
*Neutrophils, 2.06-6.49FM *10e9/L*	4.85	4.63	2.00	**9.65**	1.66	0.23
**Spearman correlation:** ***platelets(0.58), leukocytes(0.91), monocytes(0.63), hsCRP(0.45),*** *MCV(-0.29),* ***basophils%(-0.36), lymphocytes%(-0.65), monocytes%(-0.42)***						
Neutrophils %, 44-72FM	58.28	58.30	38.60	**75.00**	8.22	1.13
**Spearman correlation:** ***Platelets(0.36), leukocytes(0.44), hsCRP(0.44),*** *basophils%(-0.33),* ***lymphocytes(-0.45), lymphocytes%(-0.95)***						
*Lymphocytes, 1.19-3.35FM *10e9/L*	2.39	2.26	1.18	**4.10**	0.71	0.10
**Spearman correlation:** ***leukocytes(0.52),*** *monocytes(0.31), Ca total(0.28),* ***albumin(0.36), cholesterol(0.43), LDL-C(0.47),*** *W-H-ratio(0.34), VFL(0.28),* ***neutrophils%(-0.45),*** *mono%(-0.34)*						
Lymphocytes %, 20-46FM	29.77	29.00	14.60	**49.20**	7.88	1.08
**Spearman correlation:** *albumin(0.28), platelets(-0.29), leukocytes(-0.33),* ***neutrophils(-0.65), neutrophils%(-0.95)****, monocytes(-0.30),* ***hsCRP(-0.43)***						

*Monocytes, 0.12-0.84FM *10e9/L*	0.68	0.64	0.36	**1.24**	0.18	0.02
**Spearman correlation:** ***platelets(0.41), leukocytes(0.71), neutrophils(0.63),*** *lymphocytes0.31),* ***W-H-ratio(0.40****), lymphocytes%(-0.30)*						
Monocytes %, 2-12FM	8.48	8.20	4.80	**13.10**	1.81	0.25
**Spearman correlation:** *MCV(0.29),* ***urates(0.41), platelets(-0.36), leukocytes(-0.43), neutrophils(-0.42),*** *lymphocytes(-0.34), hsCRP(-0.30), TC(-0.33)*						
**Glucose,** *4.4-6.4FM mmol/L*	**7.18**	**6.40**	**4.30**	**19.10**	**2.30**	**0.32**
**Spearman correlation:** inorganic phosphates(0.34), **hs troponin(0.50), fT4(0.49), HOMA-IR(0.57)**						
**Urate,** *134-337F, 182-403M µmol/L*	**412.19**	**395.00**	**247.00**	**838.00**	**122.28**	**16.80**
**Spearman correlation:** *MCV(0.34),* ***monocytes%(0.41),*** *total Ca(0.32),* ***hs troponin(0.35),*** *weight(0.29),* ***SMM(0.37), BMR(0.37), platelets(-0.39),*** *HDL(-0.34), fT3(-0.30)*						
Urea, 2.8-8.3 mmol/L	6.64	6.00	3.90	**18.70**	2.58	0.35
**Spearman correlation:** ***erythrocytes(0.50), hemoglobin(0.45), hematocrit(0.41),*** *albumin(-0.31)*						
Total calcium, 2.14-2.53FM mmol/L	2.43	2.43	2.23	**2.71**	0.11	0.02
**Spearman correlation:** *lymphocytes(0.28), urates(0.32),* ***inorganic phosphates(0.51), chlorides(0.46), albumin(0.41),*** *TG(0.27)*						
Inorganic phosphates, 0.79-1.42FM mmol/L	1.03	0.93	0.74	**1.53**	0.23	0.03
**Spearman correlation:** *RDW(0.30), glucose(0.34),* ***Ca total(0.51), chlorides(0.71), hstroponin(0.51), fT4(0.35), weight(0.36), SMM(0.35), BMI(0.35), BMR(0.36), platelets(-0.36)***						
Chlorides, 97-108 mmol/L	50.55	1.35	0.84	108.00	50.91	6.99
**Spearman correlation:** ***RDW(0.42), Ca total(0.46), inorganic phosphates(0.71), hs troponin(0.44),*** *fT4(0.28),* ***weight(0.41), SMM(0.37), BMI(0.46), BMR(0.38), platelets(-0.39)***						
**Albumin,** *40.6-51.4FM g/L*	44.07	44.00	38.20	49.30	2.48	0.34
**Spearman correlation:** *hemoglobin(0.28),* ***lymphocytes(0.36),*** *lymphocytes%(0.28),* ***Ca total(0.41), fT3(0.41),*** *urea(-0.31), weight(-0.31),* ***BFM(-0.47), BMI(-0.39)***						
**hsCRP,** ≤5FM mg/L	**9.94**	**6.78**	**0.99**	**39.64**	**9.20**	**1.26**
**Spearman correlation:** ***lymphocytes%(-0.43),*** *monocytes(-0.30)*						
hs troponin I, *≤15.6F, ≤34.2M ng/L*	3.12	2.00	0.10	18.70	3.47	0.48
**Spearman correlation:** ***basophils(0.37), basophils%(0.36), glucose(0.50), urates(0.35), inorganic phosphates(0.51), chlorides(0.44), fT4(0.36),*** *HOMA-IR(0.33),* ***weight(0.37), SMM(0.46),*** *BMI(0.30),* ***BMR(0.49),*** *platelets(-0.28),* ***TC(-0.36), LDL-C(-0.36),*** *fT3(-0.33), PBF%(-0.27),* ***VFL(-0.39)***						
**TC,** *≤5FM, mmol/L*	**5.65**	**5.60**	**3.00**	**10.10**	**1.46**	**0.20**
**Spearman correlation:** *platelets(0.30),* ***lymphocytes(0.43), HDL-C(0.40), LDL-C(0.96), TG(0.41),*** *basophils%(-0.29), monocytes%(-0.33),* ***hs troponin(-0.36)***						
HDL-C, *≤1.2F, ≤1M mmol/L*	1.24	1.20	0.80	2.10	0.30	0.04

**Spearman correlation:** ***TC(0.40),*** *fT3(0.32),* ***erythrocytes(-0.36),*** *urates(-0.34), insulin(-0.33),* ***weight(-0.41), SMM(-0.44), BMR(-0.45),*** *ratio(-0.29)*						
**LDL-C,** *≤3FM mmol/L*	**3.56**	**3.20**	**1.00**	**7.70**	**1.25**	**0.17**
**Spearman correlation*****: lymphocytes(0.47), TC(0.96), TG(0.35),*** *basophils%(-0.30),* ***hs troponin(-0.36)***						
**TG,** *≤1.7FM mmol/L*	**2.02**	**2.00**	**0.80**	**4.60**	**0.82**	**0.11**
**Spearman correlation:** *erythrocytes(0.32), hematocrit(0.27), Ca total(0.27),* ***TC(0.41), LDL-C(0.35)***						
TSH, 0.35-4.94FM mIU/L	2.38	2.05	0.08	**7.69**	1.39	0.19
**Spearman correlation:** *fT4(0.27)*						
**fT4,** *9-19.5FM pmol/L*	12.80	12.60	9.32	18.57	1.91	0.26
**Spearman correlation:** ***glucose(0.49), inorganic phosphates(0.35),*** *chlorides(0.28),* ***hs troponin(0.36),*** *TSH(0.27)*						
**fT3,** *2.42-6FM, pmol/L*	4.22	4.25	2.64	5.59	0.72	0.10
**Spearman correlation:** *hemoglobin(0.33), hematocrit(0.30),* ***albumin(0.41),*** *HDL(0.32), basophils(-0.28), urates(-0.30), hs troponin(-0.33), weight(-0.34),* ***BFM(-0.37), BMI(-0.40)***						
Insulin, 2-22FM mU/L	17.40	15.10	4.30	**56.30**	8.87	1.22
**Spearman correlation:** *erythrocytes(0.39), HOMA-IR(0.90), weight(0.40), SMM(0.34), BMI(0.33), BMR(0.33), MCV(-0.56), HDL(-0.33)*						
**HOMA-IR,** *<2.5FM*	**5.85**	**4.68**	**1.40**	**19.77**	**4.06**	**0.56**
**Spearman correlation:** *RDW(0.31),* ***glucose(0.57),*** *hs troponin(0.33),* ***insulin(0.90), weight(0.41),*** *SMM(0.32), BFM(0.33),* ***BMI(0.43),*** *BMR(0.32),* ***MCV(-0.51)***						

F-female, M-male, MCV-mean corpuscular volume of red blood cells, RDW-red cell distribution width, hsCRP- high sensitivity C-reactive protein, TC-total cholesterol; in bold are levels higher than the reference range values. In bold are moderately (0.4-0.59), strongly (0.6-0.79) and very strongly (0.8-1) correlated variables.

Table S4 DNA damage parameters measured in all three assays on the group level (n=53) with all significant Spearman correlation found when compared with each assay parameters and with anthropometric and biochemical parameters

Micronucleus assay	**Mean**	**Median**	**Min**	**Max**	**SD**	**SE**
**M1**	**538.96**	**545.00**	**186.00**	**912.00**	**264.62**	**36.35**
Spearman correlation: *NDI(-0.98), M4(0.91), M3(-0.91), M2(-0.89), NB(-0.43), inorganic phosphates(0.52), chlorides(0.50), BMI(0.48), fT3(-0.45), weight(0.38), BFM(0.36), Ca total(0.38), MN(0.35), hs troponin(0.34), fT4(0.32)*						
**M2**	**316.94**	**330.00**	**84.00**	**575.00**	**156.10**	**21.44**
Spearman correlation*: M1(0.89), NDI(0.81), M4(0.72), M3(0.71), fT3(0.49), chlorides(-0.48), NB(0.45), BMI(-0.46), BFM(-0.38), Ca total(-0.33), inorganic phosphates(-0.42), weight(-0.35), MN(-0.35), hs troponin(-0.27)*						
M3	51.83	51.00	1.00	140.00	42.55	5.84
Spearman correlation: *NDI(0.93), M4(0.92), M1(-0.91), M2(0.71), inorganic phosphates(-0.55), chlorides(-0.53), BMI(-0.46), hs troponin(-0.43), fT3(0.42), weight(-0.40), Ca total(-0.40), MN(-0.39), fT4(-0.38), NB(0.35), neutrophils(0.34), neutrophils%(0.32), BFM(-0.32), SMM(-0.29), BMR(-0.29), leukocytes(0.29)*						
M4	91.87	61.00	0.00	302.00	94.59	12.99
Spearman correlation: *NDI(0.96), M3(0.92), M1(-0.91), M2(0.72), chlorides(-0.58), inorganic phosphates(-0.55), BMI(-0.54), NB(0.47), weight(-0.46), hs troponin(-0.43), Ca total(-0.41), fT3(0.40), SMM(-0.35), BFM(-0.35), BMR(-0.34), MN(-0.32), platelets(0.31), neutrophils(0.31), urates(-0.30), neutrophils%(0.29), glucose(-0.29), fT4(-0.27)*						
**NDI, ~2**	**1.70**	**1.74**	**1.09**	**2.55**	**0.48**	**0.07**
Spearman correlation: *M1(-0.98), M4(0.96), M3(0.93), M2(0.81), inorganic phosphates(-0.53), chlorides(0.52), BMI(-0.48), weight(-0.42), fT3(0.41), NB(0.40), hs troponin(-0.39), Ca total(-0.37), BFM(-0.34), fT4(-0.33), SMM(-0.32), MN(-0.32), BMR(-0.31), glucose(-0.27), neutrophils(0.27)*						
FREQ MN, 0-12.5	9.00	7.50	1.50	24.00	5.10	0.70
Spearman correlation: *inorganic phosphates(0.42), M3(-0.39), fT3(-0.39), M1(0.35), M2(-0.35), M4(-0.32), NDI(-0.32), urates(0.30), fT4(0.29), chlorides(0.27),*						
**FREQ NB, 0-5**	**5.60**	**3.50**	**0.00**	**21.00**	**5.12**	**0.70**
Spearman correlation: *M4(0.47), chlorides(-0.46), M2(0.45), Ca total(-0.43), M1(-0.43), NDI(0.40), BMI(-0.39), fT3(0.39), W-H-ratio(0.38), M3(0.35), TG(-0.34), weight(-0.31), inorganic phosphates(-0.29), hs troponin(-0.28), mean comet(-0.27)*						
FREQ NPB, 0-10	5.74	4.50	0.50	**17.00**	4.31	0.59
Spearman correlation: *urates(0.36), MCV(0.29)*						
**APOPTOSIS, 0-7%**	**7.91**	**7.00**	**0.00**	**42.00**	**7.02**	**0.96**
Spearman correlation: *mean comet(0.36), basophils(0.30), necrosis(0.29), glucose(0.27), albumin(0.28)*						
NECROSIS, 0-9%	2.79	0.00	0.00	**33.00**	7.45	1.02
Spearman correlation: *TSH(0.53), inorganic phosphates(0.37), glucose(0.35), neutrophils%(0.34), limfo%(-0.33), fT4(0.33), RDW(0.31), hs troponin(0.30), chlorides(0.29), apoptosis(0.29), neutrophils(0.28)*						
Alkaline comet assay						
**Mean comet (TI), 0-9%**	**10.51**	**10.04**	**4.50**	**18.27**	**4.00**	**0.55**
Spearman correlation: *TG(0.43), chlorides(0.41), apoptosis(0.36), glucose(0.35), inorganic phosphates(0.30), HDL(0.30)*						
Fpg alkaline comet assay						
**Net Fpg (TI), 0%**	**6.28**	**4.45**	**0.00**	**31.59**	**6.20**	**0.85**
Spearman correlation: *basophils(-0.27), basophils%(-0.27)*						

SD-standard deviation, SE- standard error, MN-micronuclei, NB- nuclear buds, NPB-nucleoplasmic bridges. TI-tail intensity (%DNA in comet tail), Net Fpg- TI% of oxidative DNA damage

Table S5. Division of the entire group according to sex and the nutrient intake and food group intake by FFQ questionnaire, together with anthropometric, biochemical and DNA damage parameters

	**Female, N=36**						**Male, N=17**					
**Variable**	**Mean**	**Median**	**Min**	**Max**	**SD**	**SE**	**Mean**	**Median**	**Min**	**Max**	**SD**	**SE**
Alpha carotene (mcg)	**154.23**	**106.50**	**6.80**	**994.71**	**172.99**	**28.83**	113.47	95.39	4.62	553.12	134.91	32.72
Alcohol (g)	3.66	0.64	0.00	43.75	9.03	1.51	**5.66**	**2.03**	**0.00**	**34.79**	**10.10**	**2.45**
Beta carotene (mcg)	**1959.72**	**1671.77**	**301.25**	**5381.19**	**1289.65**	**214.94**	1614.62	1206.41	608.99	4746.36	1090.60	264.51
Calcium (mg)	**711.53**	**570.82**	**180.57**	**3734.16**	**601.75**	**100.29**	513.624	482.98	219.54	1184.92	239.69	58.13
Carotene - total (carotene equivalents) (mcg)	**2243.28**	**1834.33**	**359.58**	**5945.25**	**1454.70**	**242.45**	1895.74	1419.29	638.77	5108.67	1213.30	294.27
Carbohydrate - total (g)	**250.14**	**185.78**	**54.23**	**1771.66**	**285.21**	**47.53**	184.49	178.00	69.92	390.24	79.79	19.35
Cholesterol (mg)	**345.81**	**245.63**	**124.05**	**2284.20**	**359.95**	**59.99**	277.87	265.58	118.47	617.10	117.14	28.41
Chloride (mg)	**3930.99**	**3224.73**	**1214.11**	**18279.11**	**2944.35**	**490.73**	3380.50	3088.06	978.96	7446.90	1453.30	352.48
Copper (mg)	**1.73**	**1.26**	**0.39**	**11.89**	**1.98**	**0.33**	1.61	1.40	0.71	3.43	0.74	0.18
**Englyst Fibre - Non Starch Polysaccharides (NSP) (g)**	**1182.64**	**14.73**	**3.81**	**42003.00**	**6997.78**	**1166.30**	12.88	11.55	6.94	27.28	5.84	1.41
Iron (mg)	**11.76**	**9.72**	**3.71**	**54.90**	**8.60**	**1.43**	10.06	9.29	5.01	17.62	3.51	0.85
Total folate (mcg)	**253.93**	**218.57**	**73.20**	**726.66**	**126.98**	**21.16**	234.45	189.21	121.49	432.60	96.55	23.42
Carbohydrate - fructose (g)	**23.85**	**15.98**	**4.82**	**110.14**	**22.23**	**3.71**	18.30	16.24	5.48	46.92	10.18	2.47
Carbohydrate - galactose (g)	**0.74**	**0.30**	**0.00**	**4.41**	**1.03**	**0.17**	0.46	0.27	0.00	1.44	0.48	0.12
Carbohydrate - glucose (g)	**24.04**	**16.58**	**5.36**	**125.52**	**22.91**	**3.82**	17.60	15.62	4.90	40.93	8.77	2.13
Carbohydrate - glucose (mcg)	**101.29**	**74.25**	**29.85**	**662.59**	**106.01**	**17.67**	71.42	65.78	31.57	194.69	38.12	9.25
Potassium (mg)	**3333.03**	**2723.92**	**1155.01**	**12996.94**	**2142.83**	**357.14**	2753.50	2542.73	1376.50	4799.77	1087.81	263.83
Energy_kcal	**2056.28**	**1619.59**	**655.90**	**12727.57**	**1992.29**	**332.05**	1475.07	1425.12	478.76	2905.58	593.89	144.04
Energy_kj	**8432.35**	**6408.36**	**2761.78**	**53529.71**	**8403.83**	**1400.64**	6209.18	5987.14	2020.33	12230.48	2498.94	606.08

Carbohydrate - lactose (g)	**5.83**	**4.15**	**0.23**	**45.08**	**7.64**	**1.27**	2.81	2.62	0.72	8.49	1.92	0.47
Carbohydrate - maltose (g)	**2.91**	**1.26**	**0.17**	**24.30**	**4.39**	**0.73**	1.54	1.27	0.06	4.99	1.19	0.29
Magnesium (mg)	**297.29**	**258.62**	**100.45**	**1139.36**	**192.66**	**32.11**	228.84	248.76	104.81	378.51	84.24	20.43
Manganese (mg)	**3.53**	**2.83**	**0.71**	**14.70**	**2.78**	**0.46**	2.55	2.72	1.12	4.06	0.98	0.24
Sodium (mg)	**2730.91**	**2190.74**	**800.92**	**13993.37**	**2214.70**	**369.12**	2319.06	2202.57	583.67	5050.05	990.96	240.34
Niacin (mg)	**25.44**	**22.23**	**9.13**	**81.92**	**15.26**	**2.54**	22.74	22.24	7.36	38.59	9.27	2.25
Phosphorus (mg)	**1355.63**	**1135.51**	**485.18**	**6624.44**	**1033.30**	**172.22**	1061.37	1060.01	453.21	1872.52	403.72	97.92
Protein (g)	**92.28**	**77.14**	**32.18**	**363.30**	**60.50**	**10.08**	74.36	70.97	25.83	142.17	29.92	7.26
Vitamin A - retinol (mcg)	1329.84	851.04	38.37	14070.95	2478.92	413.15	**1593.80**	**1515.88**	**54.90**	**5250.17**	**1480.40**	**359.05**
Vitamin A - retinol equivalents (mcg)	1705.21	1079.89	253.20	14829.73	2560.58	426.76	**1912.77**	**1839.53**	**161.84**	**5370.13**	**1413.50**	**342.82**
Vitamin B2 - riboflavin (mg)	**1.54**	**1.10**	**0.605**	**8.31**	**1.34**	**0.22**	1.44	1.37	0.71	2.34	0.55	0.13
Selenium (mcg)	**74.99**	**68.77**	**29.95**	**263.07**	**43.57**	**7.26**	61.945	57.58	22.55	103.99	23.04	5.59
Carbohydrate - starch (g)	**121.54**	**89.39**	**13.84**	**800.47**	**126.53**	**21.09**	95.79	91.68	33.43	196.79	45.07	10.93
Carbohydrate - sucrose (g)	**61.02**	**27.66**	**9.77**	**605.67**	**104.71**	**17.45**	33.88	29.84	14.17	105.51	20.90	5.07
Vitamin B1 - thiamin (mg)	**67.84**	**1.16**	**0.47**	**2395.00**	**398.94**	**66.49**	1.33	1.53	0.56	2.29	0.56	0.14
Nitrogen (g)	**15.02**	**12.56**	**5.30**	**59.56**	**9.89**	**1.65**	12.02	11.31	4.14	22.85	4.82	1.17
Carbohydrate - sugars (total) (g)	**122.59**	**75.63**	**31.33**	**939.79**	**160.57**	**26.76**	80.44	68.63	33.95	189.50	40.01	9.71
Vitamin B12 - cobalamin (mcg)	7.36	5.65	1.65	52.37	8.71	1.45	**8.16**	**7.38**	**1.45**	**19.61**	**5.24**	**1.27**
Vitamin B6 - pyridoxine (mg)	**1.95**	**1.59**	**0.66**	**7.14**	**1.19**	**0.20**	1.65	1.43	0.81	3.51	0.76	0.18
Vitamin C - ascorbic acid (mg)	**122.00**	**69.21**	**18.41**	**1111.13**	**182.60**	**30.43**	85.25	76.94	38.37	161.25	39.29	9.53
Vitamin D - ergocalciferol (mcg)	**2.91**	**2.01**	**0.99**	**22.85**	**3.68**	**0.61**	2.61	2.45	0.81	5.22	1.48	0.36

Vitamin E - alpha tocopherol equivalents (mg)	**11.20**	**8.57**	**3.34**	**75.19**	**12.17**	**2.03**	8.56	7.84	3.15	18.10	4.51	1.09
Zinc (mg)	**9.88**	**8.54**	**3.75**	**40.46**	**6.36**	**1.06**	8.30	8.14	2.89	20.35	3.95	0.96
Fat - total (g)	**74.64**	**57.03**	**20.14**	**513.83**	**82.48**	**13.75**	49.60	47.48	12.57	109.32	22.62	5.49
Monounsaturated fatty acids (MUFA - total) (g)	**29.40**	**22.55**	**7.93**	**210.71**	**34.76**	**5.79**	18.65	18.56	4.64	40.11	8.65	2.10
Polyunsaturated fatty acids (PUFA - total) (g)	**11.83**	**8.94**	**3.60**	**68.10**	**11.28**	**1.88**	8.65	7.97	2.60	14.63	3.41	0.83
Saturated fatty acids (SFA - total) (g)	**26.34**	**20.69**	**5.88**	**186.60**	**29.65**	**4.94**	17.36	15.82	3.70	44.22	9.12	2.21
Alcoholic beverages (g)	20.38	3.50	0.00	138.00	32.21	5.37	**41.85**	**9.89**	**0.00**	**316.00**	**79.87**	**19.37**
Cereals and cereal products (g)	**303.87**	**199.07**	**0.56**	**2585.28**	**432.87**	**72.14**	182.06	171.05	36.19	448.68	105.20	25.51
Eggs and egg dishes (g)	**16.54**	**17.50**	**0.00**	**50.00**	**13.73**	**2.29**	15.74	21.50	3.50	50.00	11.91	2.89
Fats and oils (g)	**13.96**	**6.02**	**0.00**	**177.12**	**31.82**	**5.30**	8.84	8.32	0.42	24.10	7.07	1.72
Fish & fish products (g)	26.39	25.66	0.00	85.40	17.31	2.88	**29.68**	**22.40**	**0.00**	**101.00**	**22.97**	**5.57**
Fruit (g)	**213.56**	**155.28**	**17.50**	**1477.80**	**274.94**	**45.82**	122.19	124.75	43.65	269.80	65.69	15.93
Meat and meat products (g)	**165.39**	**138.54**	**42.42**	**613.13**	**121.09**	**20.18**	145.08	132.24	20.65	318.23	75.46	18.30
Milk and milk products (g)	**112.82**	**76.74**	**0.00**	**423.20**	**112.79**	**18.80**	77.12	78.88	5.60	136.18	46.61	11.31
Non-alcoholic beverages (g)	**642.48**	**517.40**	**9.48**	**1791.10**	**454.91**	**75.82**	619.03	486.60	28.00	1494.14	375.53	91.08
Nuts and seeds (g)	**17.46**	**3.43**	**0.00**	**137.66**	**30.32**	**5.05**	4.80	2.10	0.00	30.00	8.51	2.06
Potatoes (g)	88.10	77.69	0.00	392.69	74.75	12.46	**98.68**	**71.39**	**17.57**	**317.18**	**78.08**	**18.94**
Soups & sauces (g)	90.68	77.20	0.00	280.40	69.08	11.51	**111.31**	**90.20**	**0.00**	**377.90**	**89.76**	**21.77**
Sugars (g)	**60.74**	**25.16**	**0.00**	**530.50**	**97.79**	**16.30**	28.81	15.77	2.58	120.46	30.29	7.35
preserves and snacks (g)	**229.23**	**226.23**	**20.64**	**747.81**	**144.99**	**24.17**	218.70	199.34	79.82	539.04	133.51	32.38
Vegetables	**4.58**	**5.00**	**0.00**	**5.00**	**1.40**	**0.23**	3.82	5.00	0.00	5.00	2.19	0.53

DII	1.92	2.17	-3.37	6.00	2.19	0.37	**2.36**	**2.32**	**-1.24**	**6.73**	**2.81**	**0.68**
hs troponin I	2.41	1.50	0.10	18.70	3.35	0.56	**4.64**	**4.00**	**1.00**	**14.10**	**3.33**	**0.81**
TC	**5.94**	**5.90**	**3.20**	**10.10**	**1.53**	**0.26**	5.04	5.10	3.00	7.20	1.11	0.27
HDL-C	**1.33**	**1.40**	**0.80**	**2.10**	**0.30**	**0.05**	1.05	1.00	0.80	1.40	0.19	0.05
LDL-C	**3.76**	**3.65**	**1.80**	**7.70**	**1.33**	**0.22**	3.13	3.10	1.00	4.90	0.98	0.24
TG	1.93	1.90	0.80	4.60	0.77	0.13	**2.22**	**2.20**	**0.80**	**3.70**	**0.91**	**0.22**
TSH	**2.68**	**2.14**	**1.00**	**7.69**	**1.51**	**0.25**	1.76	1.69	0.08	3.02	0.83	0.20
fT4	**12.91.**	**12.53**	**10.19**	**18.57**	**1.95**	**0.32**	12.56	12.77	9.32	16.37	1.86	0.45
fT3	**4.23**	**4.28**	**2.64**	**5.51**	**0.69**	**0.12**	4.19	4.21	2.71	5.59	0.80	0.19
Insulin	16.40	14.10	6.80	31.40	6.54	1.09	**19.52**	**17.70**	**4.30**	**56.30**	**12.46**	**3.02**
HOMA-IR	**29.28**	**26.11**	**3.28**	**113.40**	**25.05**	**4.18**	25.05	5.40	1.40	127.21	40.91	9.92
Weight	119.86	120.50	95.30	145.30	14.71	2.45	**140.95**	**134.90**	**118.70**	**183.60**	**20.37**	**4.94**
SMM	32.84	33.20	23.80	42.70	4.27	0.71	**46.65**	**46.80**	**33.00**	**56.20**	**6.12**	**1.48**
BFM	**61.35**	**62.10**	**46.00**	**78.40**	**9.10**	**1.52**	59.57	59.70	40.40	90.30	15.74	3.82
PBF%	**50.39**	**50.90**	**43.60**	**56.30**	**3.55**	**0.59**	42.75	41.80	30.30	64.50	8.46	2.05
BMI	44.48	43.30	35.40	57.50	5.59	0.93	**44.78**	**43.80**	**35.70**	**54.80**	**6.32**	**1.53**
BMR	1633.83	1641.50	1299.00	1990.00	156.04	26.01	**2127.82**	**2120.00**	**1647.00**	**2462.00**	**213.70**	**51.83**
ratio	1.03	1.04	0.70	1.22	0.10	0.02	**1.10**	**1.13**	**0.84**	**1.27**	**0.11**	**0.03**
VFL	**19.89**	**20.00**	**16.00**	**20.00**	**0.67**	**0.11**	19.53	20.00	16.00	20.00	1.01	0.24
M1	508.39	412.50	186.00	912.00	265.63	44.27	**603.71**	**596.00**	**197.00**	**883.00**	**258.23**	**62.63**
M2	**328.03**	**352.00**	**84.00**	**569.00**	**152.08**	**25.35**	293.47	263.00	111.00	575.00	166.54	40.39

M3	**58.00**	**55.00**	**1.00**	**140.00**	**43.87**	**7.31**	38.76	25.00	2.00	113.00	37.50	9.10
M4	**105.58**	**73.50**	**0.00**	**302.00**	**102.02**	**17.00**	62.82	14.00	1.00	214.00	70.64	17.13
NDI	**1.76**	**1.81**	**1.09**	**2.55**	**0.50**	**0.08**	1.56	1.42	1.12	2.32	0.43	0.10
FREQ MN TOTAL	8.82	7.50	1.50	24.00	5.47	0.91	**9.38**	**7.50**	**4.50**	**17.50**	**4.37**	**1.06**
FREQ NB TOTAL	**5.67**	**3.50**	**0.50**	**21.00**	**4.95**	**0.83**	5.47	3.50	0.00	20.50	5.64	1.37
FREQ NPB TOTAL	5.26	3.25	0.50	17.00	4.23	0.71	**6.74**	**6.50**	**1.00**	**16.00**	**4.42**	**1.07**
APOPTOSIS	6.78	5.50	0.00	26.00	5.56	0.93	**10.29**	**8.00**	**2.00**	**42.00**	**9.14**	**2.22**
NECROSIS	2.14	0.00	0.00	29.00	6.28	1.05	**4.18**	**0.00**	**0.00**	**33.00**	**9.55**	**2.32**
Net Fpg, TI	**6.56**	**4.34**	**0.00**	**31.59**	**6.86**	**1.14**	5.68	4.93	0.00	16.66	4.64	1.12
TI	10.15	9.52	4.50	18.27	4.34	0.72	**11.26**	**10.53**	**6.79**	**17.88**	**3.15**	**0.76**
Age	50.30	49.17	27.00	67.00	12.00	2.00	**52.96**	**55.00**	**26.35**	**68.00**	**10.79**	**2.62**

Table S6. The differences in the nutrient intake and food group intake by the FFQ questionnaire, together with anthropometric, biochemical and DNA damage parameters observed in two age groups: ≥60 and < 60 years with higher values in bold

	**≥60, n=18**						**<60, n=35**					
**Variable**	**Mean**	**Median**	**Min**	**Max**	**SD**	**SE**	**Mean**	**Median**	**Min**	**Max**	**SD**	**SE**
Alpha carotene (mcg)	107.81	104.31	6.80	201.21	65.64	15.47	158.31	105.85	4.62	994.71	192.06	32.46
Alcohol (g)	1.42	0.49	0.00	7.29	2.24	0.53	**5.79**	**0.92**	**0.00**	**43.75**	**11.14**	**1.88**
Beta carotene (mcg)	1713.14	1470.23	672.48	4899.43	1011.81	238.49	**1918.92**	**1634.15**	**301.25**	**5381.19**	**1336.27**	**225.87**
Calcium (mg)	512.33	470.21	180.57	1159.42	286.55	67.54	**717.85**	**568.80**	**219.54**	**3734.16**	**597.72**	**101.03**
Carotene - total (carotene equivalents) (mcg)	1926.08	1649.10	939.15	4991.14	1050.48	247.60	**2237.60**	**1820.92**	**359.581**	**5945.25**	**1525.28**	**257.82**
Carbohydrate - total (g)	172.76	160.18	69.92	407.87	79.72	18.79	**258.05**	**211.08**	**54.23**	**1771.66**	**287.18**	**48.54**
Cholesterol (mg)	281.25	284.15	135.62	802.80	153.52	36.19	**346.00**	**246.26**	**118.47**	**2284.20**	**357.98**	**60.51**
Chloride (mg)	2932.71	2831.96	978.96	4849.94	1276.83	300.95	**4177.02**	**3408.24**	**1528.34**	**18279.11**	**2943.56**	**497.55**
Copper (mg)	1.38	1.29	0.74	2.96	0.54	0.13	**1.86**	**1.36**	**0.39**	**11.89**	**2.02**	**0.34**
Englyst Fibre - Non Starch Polysaccharides (NSP) (g)	12.96	13.082	6.943	20.97	4.301	1.0138	**1216.03**	**14.04**	**3.81**	**42003.00**	**7097.04**	**1199.62**
Iron (mg)	9.21	8.07	5.33	14.99	2.86	0.67	**12.25**	**10.70**	**3.71**	**54.90**	**8.69**	**1.47**
Total folate (mcg)	222.99	218.57	117.57	372.99	69.77	16.44	**260.38**	**199.18**	**73.20**	**726.66**	**134.88**	**22.80**
Carbohydrate - fructose (g)	18.66	16.25	6.41	47.21	9.30	2.19	**23.82**	**15.88**	**4.82**	**110.14**	**22.70**	**3.84**
Carbohydrate - galactose (g)	0.63	0.19	0.00	2.64	0.83	0.20	**0.66**	**0.37**	**0.00**	**4.41**	**0.93**	**0.16**
Carbohydrate - glucose (g)	17.42	16.05	6.77	46.54	9.14	2.15	**24.31**	**15.68**	**4.90**	**125.52**	**23.07**	**3.90**
Carbohydrate - glucose (mcg)	72.94	65.05	29.85	152.54	35.89	8.46	**101.37**	**70.91**	**31.57**	**662.59**	**107.84**	**18.23**
Potassium (mg)	2625.24	2593.03	1366.62	4640.72	874.81	206.20	**3415.55**	**2732.09**	**1155.01**	**12996.94**	**2190.14**	**370.20**
Energy_kcal	1396.43	1333.21	478.76	2536.29	543.87	128.19	**2113.33**	**1650.74**	**844.57**	**12727.57**	**2009.75**	**339.71**

Energy_kj	5881.17	5622.39	2020.32	10707.78	2291.68	540.15	**8664.56**	**6869.45**	**3563.39**	**53529.71**	**8484.36**	**1434.12**
Carbohydrate - lactose (g)	3.48	1.93	0.23	9.65	3.33	0.79	**5.57**	**3.54**	**0.96**	**45.08**	**7.61**	**1.29**
Carbohydrate - maltose (g)	1.41	0.93	0.06	6.30	1.52	0.36	**3.02**	**1.86**	**0.14**	**24.30**	**4.36**	**0.74**
Magnesium (mg)	229.96	249.20	104.81	376.93	79.34	18.70	**298.67**	**263.62**	**100.45**	**1139.36**	**195.82**	**33.10**
Manganese (mg)	2.57	2.55	1.12	4.06	0.97	0.23	**3.54**	**2.77**	**0.71**	**14.70**	**2.81**	**0.48**
Sodium (mg)	2024.83	1912.82	583.67	3279.56	888.33	209.38	**2893.99**	**2235.46**	**1106.84**	**13993.37**	**2215.90**	**374.56**
Niacin (mg)	22.44	22.12	7.36	65.05	12.25	2.89	**25.68**	**22.24**	**9.13**	**81.92**	**14.28**	**2.41**
Phosphorus (mg)	1063.72	1021.24	453.21	2312.87	456.64	107.63	**1362.83**	**1109.68**	**528.77**	**6624.44**	**1034.25**	**174.82**
Protein (g)	77.65	74.96	25.83	216.21	41.24	9.72	**91.09**	**75.40**	**38.75**	**363.30**	**58.17**	**9.83**
Vitamin A - retinol (mcg)	1151.88	1033.91	38.37	5004.15	1148.51	270.71	**1549.57**	**894.91**	**54.90**	**14070.95**	**2581.85**	**436.41**
Vitamin A - retinol equivalents (mcg)	1474.40	1487.65	272.41	5172.92	1132.80	267.00	**1924.73**	**1374.06**	**161.84**	**14829.73**	**2644.19**	**446.95**
Vitamin B2 - riboflavin (mg)	1.28	1.12	0.61	2.47	0.53	0.13	**1.62**	**1.161**	**0.61**	**8.31**	**1.34**	**0.23**
Selenium (mcg)	64.43	67.26	22.55	151.51	28.87	6.80	**74.09**	**65.16**	**29.95**	**263.07**	**42.58**	**7.20**
Carbohydrate - starch (g)	90.17	79.03	33.43	237.32	47.25	11.14	**125.17**	**91.68**	**13.84**	**800.47**	**126.96**	**21.46**
Carbohydrate - sucrose (g)	32.91	22.75	9.77	160.22	33.78	7.96	**62.29**	**32.20**	**12.11**	**605.67**	**104.26**	**17.62**
Vitamin B1 - thiamin (mg)	1.10	1.09	0.56	1.74	0.36	0.09	**69.85**	**1.16**	**0.47**	**2395.00**	**404.58**	**68.39**
Nitrogen (g)	12.59	12.16	4.14	34.78	6.64	1.56	**14.81**	**12.17**	**6.36**	**59.56**	**9.54**	**1.61**
Carbohydrate - sugars (total) (g)	77.47	67.93	31.33	273.17	53.22	12.55	**125.32**	**78.11**	**33.95**	**939.79**	**160.25**	**27.09**
Vitamin B12 - cobalamin (mcg)	6.54	6.15	2.61	18.15	3.50	0.83	**8.18**	**6.21**	**1.45**	**52.37**	**9.17**	**1.55**
Vitamin B6 - pyridoxine (mg)	1.57	1.56	0.77	3.71	0.65	0.15	**2.00**	**1.57**	**0.66**	**7.14**	**1.22**	**0.21**
Vitamin C - ascorbic acid (mg)	84.16	79.79	38.37	207.07	40.83	9.62	**123.61**	**68.69**	**18.41**	**1111.13**	**184.75**	**31.23**
Vitamin D - ergocalciferol (mcg)	2.06	1.87	1.01	4.52	0.95	0.22	**3.20**	**2.28**	**0.81**	**22.85**	**3.76**	**0.64**

Vitamin E - alpha tocopherol equivalents (mg)	7.86	7.84	3.15	15.78	3.34	0.79	**11.64**	**8.23**	**3.34**	**75.19**	**12.40**	**2.10**
Zinc (mg)	7.90	7.68	2.89	17.55	3.35	0.79	**10.13**	**8.76**	**3.75**	**40.46**	**6.52**	**1.10**
Fat - total (g)	47.58	43.48	12.57	85.28	21.12	4.98	**76.39**	**56.78**	**22.78**	**513.83**	**83.33**	**14.09**
Monounsaturated fatty acids (MUFA - total) (g)	18.08	16.72	4.64	30.35	7.68	1.81	**30.00**	**21.34**	**8.11**	**210.71**	**35.20**	**5.95**
Polyunsaturated fatty acids (PUFA - total) (g)	8.70	7.98	2.60	15.13	3.76	0.89	**11.89**	**9.36**	**3.60**	**68.10**	**11.36**	**1.92**
Saturated fatty acids (SFA - total) (g)	16.02	14.01	3.70	38.08	8.81	2.08	**27.29**	**19.88**	**7.57**	**186.60**	**29.80**	**5.04**
Alcoholic beverages (g)	7.26	2.42	0.00	50.00	12.37	2.92	**37.55**	**19.11**	**0.00**	**316.00**	**61.89**	**10.46**
Cereals and cereal products (g)	210.82	169.36	21.18	975.65	210.87	49.70	**292.56**	**188.91**	**0.56**	**2585.28**	**422.57**	**71.43**
Eggs and egg dishes (g)	**17.06**	**21.50**	**3.50**	**50.00**	**12.90**	**3.04**	15.89	17.50	0.00	50.00	13.32	2.25
Fats and oils (g)	6.37	6.37	0.00	14.52	4.59	1.08	**15.37**	**6.51**	**0.21**	**177.12**	**32.18**	**5.44**
Fish & fish products (g)	**27.73**	**27.30**	**0.00**	**54.74**	**15.23**	**3.59**	27.30	20.23	0.00	101.00	21.08	3.56
Fruit (g)	155.24	138.08	43.65	326.05	84.76	19.98	**199.17**	**129.05**	**17.50**	**1477.80**	**280.03**	**47.33**
Meat and meat products (g)	141.94	118.32	20.65	613.13	124.81	29.42	**167.59**	**142.58**	**50.61**	**533.97**	**99.47**	**16.81**
Milk and milk products (g)	96.88	48.23	0.00	386.22	110.41	26.02	**103.68**	**81.96**	**4.06**	**423.20**	**91.87**	**15.53**
Non-alcoholic beverages (g)	627.07	517.40	240.10	1791.10	379.10	89.36	**639.01**	**503.00**	**9.48**	**1752.20**	**455.71**	**77.03**
Nuts and seeds (g)	9.80	3.43	0.00	30.00	10.59	2.50	**15.25**	**2.10**	**0.00**	**137.66**	**31.12**	**5.26**
Potatoes (g)	74.90	74.69	17.50	148.94	36.86	8.69	**100.02**	**74.54**	**0.00**	**392.69**	**88.09**	**14.89**
Soups & sauces (g)	88.28	84.30	0.00	216.10	58.67	13.83	**101.94**	**76.40**	**0.00**	**377.90**	**84.04**	**14.21**
Sugars (g)	25.04	11.55	0.00	133.52	40.56	9.56	**63.58**	**37.49**	**0.00**	**530.50**	**96.34**	**16.28**
preserves and snacks (g)	198.31	210.83	26.10	378.74	101.73	23.98	**240.02**	**213.05**	**20.64**	**747.81**	**155.83**	**26.34**
Vegetables	**4.44**	**5.00**	**0.00**	**5.00**	**1.62**	**0.38**	4.29	5.00	0.00	5.00	1.78	0.30
DII	**2.46**	**2.07**	**-3.37**	**6.73**	**2.53**	**0.60**	1.86	2.22	-3.08	6.52	2.33	0.39

hs troponin I	**4.46**	**4.00**	**0.80**	**18.70**	**4.14**	**0.98**	2.44	1.60	0.10	14.10	2.91	0.49
TC (total cholesterol)	5.40	5.50	3.00	7.80	1.23	0.29	**5.78**	**5.90**	**3.20**	**10.10**	**1.57**	**0.27**
HDL-C	**1.35**	**1.40**	**0.80**	**2.10**	**0.36**	**0.08**	1.18	1.20	0.80	1.80	0.26	0.04
LDL-C	3.26	3.15	1.00	5.20	1.15	0.27	**3.72**	**3.50**	**2.00**	**7.70**	**1.29**	**0.22**
TG	1.88	1.75	1.20	3.60	0.66	0.16	**2.10**	**2.20**	**0.80**	**4.60**	**0.89**	**0.15**
TSH	**2.31**	**1.71**	**0.08**	**7.69**	**1.81**	**0.43**	2.42	2.21	0.75	5.74	1.15	0.20
fT4	**13.62**	**13.10**	**10.43**	**18.57**	**2.13**	**0.50**	12.38	12.16	9.32	15.84	1.66	0.28
fT3	4.04	4.17	2.64	5.51	0.83	0.20	**4.31**	**4.25**	**2.71**	**5.59**	**0.65**	**0.11**
Insulin	16.14	13.65	9.80	30.90	6.34	1.49	**18.05**	**16.30**	**4.30**	**56.30**	**9.95**	**1.68**
HOMA-IR	18.51	9.40	2.80	111.30	25.37	5.98	**32.77**	**23.24**	**1.40**	**127.21**	**32.35**	**5.47**
Weight	124.32	124.05	95.30	177.50	19.74	4.65	**127.80**	**126.70**	**95.90**	**183.60**	**19.24**	**3.25**
BFM	35.01	33.75	23.80	53.10	7.28	1.72	**38.43**	**35.80**	**26.90**	**56.20**	**8.39**	**1.42**
Fat mass	**62.04**	**63.40**	**40.50**	**85.20**	**10.85**	**2.56**	60.13	59.70	40.40	90.30	11.95	2.02
PBF%	49.11	50.60	34.10	56.30	5.18	1.22	**47.34**	**49.40**	**30.30**	**64.50**	**7.21**	**1.22**
BMI	**46.12**	**45.70**	**37.30**	**56.10**	**5.29**	**1.25**	43.78	42.50	35.40	57.50	5.92	1.00
BMR	1715.44	1687.00	1299.00	2363.00	262.45	61.86	**1831.80**	**1750.00**	**1418.00**	**2462.00**	**300.43**	**50.78**
W-H-ratio	1.02	1.04	0.70	1.26	0.13	0.03	**1.07**	**1.07**	**0.84**	**1.27**	**0.09**	**0.02**
VFL	19.72	20.00	16.00	20.00	0.96	0.23	**19.80**	**20.00**	**16.00**	**20.00**	**0.72**	**0.12**
M1	**604.94**	**742.50**	**208.00**	**912.00**	**272.52**	**64.23**	505.03	423.00	186.00	890.00	257.83	43.58
M2	290.89	220.50	84.00	561.00	170.08	40.09	**330.34**	**348.00**	**102.00**	**575.00**	**149.20**	**25.22**
M3	38.17	19.00	2.00	113.00	39.95	9.42	**58.86**	**58.00**	**1.00**	**140.00**	**42.67**	**7.21**
M4	66.00	17.50	1.00	271.00	80.34	18.94	**105.17**	**80.00**	**0.00**	**302.00**	**99.60**	**16.84**

NDI	1.57	1.31	1.09	2.44	0.46	0.11	**1.76**	**1.76**	**1.12**	**2.55**	**0.48**	**0.08**
FREQ MN TOTAL	**11.39**	**10.00**	**3.00**	**24.00**	**6.09**	**1.44**	7.77	7.00	1.50	19.00	4.09	0.69
FREQ NB TOTAL	**6.64**	**3.75**	**0.50**	**21.00**	**6.50**	**1.53**	5.07	3.50	0.00	18.00	4.27	0.72
FREQ NPB TOTAL	**6.14**	**5.50**	**0.50**	**16.00**	**4.45**	**1.05**	5.53	4.00	1.00	17.00	4.28	0.72
APOPTOSIS	**8.61**	**7.00**	**0.00**	**26.00**	**6.95**	**1.64**	7.54	6.00	1.00	42.00	7.13	1.21
NECROSIS	**4.44**	**0.00**	**0.00**	**33.00**	**9.19**	**2.17**	1.94	0.00	0.00	29.00	6.36	1.08
Net Fpg, TI	**7.46**	**4.59**	**0.00**	**31.59**	**8.74**	**2.06**	5.67	4.46	0.00	15.06	4.42	0.75
TI	**10.73**	**11.27**	**4.55**	**18.27**	**4.19**	**0.99**	10.39	10.04	4.50	17.88	3.96	0.67
Age	**63.38**	**63.82**	**60.00**	**68.00**	**2.51**	**0.59**	44.86	45.80	26.35	59.00	9.08	1.53

Table S7. The nutrient and food group intake (based on the FFQ questionnaire), together with anthropometric, biochemical and DNA damage parameters observed after the division of the study group into two age groups: >50 and ≤50 years.

	**>50 (n=27)**						**≤50 years (n=26)**					
**Variable**	**Mean**	**Median**	**Min**	**Max**	**SD**	**SE**	**Mean**	**Median**	**Min**	**Max**	**SD**	**SE**
**Alpha carotene Alcohol (mcg)**	106.88	102.46	6.80	203.51	68.09	13.10	**176.75**	**106.08**	**4.62**	**994.71**	**216.57**	**42.47**
**Alcohol (g)**	2.85	0.51	0.00	34.79	6.85	1.32	**5.82**	**2.02**	**0.00**	**43.75**	**11.31**	**2.22**
**Beta carotene (mcg)**	1666.59	1549.72	608.99	4899.43	905.76	174.31	**2038.49**	**1611.24**	**301.25**	**5381.19**	**1489.37**	**292.09**
**Calcium (mg)**	532.60	479.50	180.57	1238.94	284.89	54.83	**767.94**	**570.08**	**263.16**	**3734.16**	**669.37**	**131.27**
**Carotene - total (carotene equivalents) (mcg)**	1899.38	1829.30	716.80	4991.14	959.65	184.69	**2373.17**	**1799.58**	**359.58**	**5945.25**	**1698.19**	**333.04**
**Carbohydrate - total (g)**	176.87	161.47	69.92	407.87	75.16	14.47	**283.31**	**212.85**	**54.23**	**1771.66**	**328.74**	**64.47**
**Cholesterol (mg)**	282.91	284.40	118.47	802.80	139.59	26.86	**366.70**	**240.45**	**124.05**	**2284.20**	**410.27**	**80.46**
**Chloride (mg)**	3163.99	2954.54	978.96	5274.23	1283.45	247.00	**4367.56**	**3406.61**	**1528.34**	**18279.11**	**3338.86**	**654.80**
**Copper (mg)**	1.43	1.31	0.74	3.43	0.62	0.12	**1.97**	**1.32**	**0.39**	**11.89**	**2.30**	**0.45**
**Englyst Fibre - Non Starch Polysaccharides (NSP) (g)**	13.21	14.06	6.94	20.97	4.12	0.79	**1632.21**	**13.09**	**3.81**	**42003.00**	**8234.07**	**1614.83**
**Iron (mg)**	9.52	9.29	5.01	14.99	2.84	0.55	**12.98**	**10.11**	**3.71**	**54.90**	**9.90**	**1.94**
**Total folate (mcg)**	229.06	221.56	117.57	372.99	73.27	14.10	**267.02**	**190.41**	**73.20**	**726.66**	**149.55**	**29.33**
**Carbohydrate - fructose (g)**	20.16	16.43	6.41	47.21	10.09	1.94	**24.05**	**14.00**	**4.82**	**110.14**	**25.66**	**5.03**
**Carbohydrate - galactose (g)**	0.60	0.25	0.00	2.64	0.74	0.14	**0.71**	**0.42**	**0.00**	**4.41**	**1.04**	**0.20**
**Carbohydrate - glucose (g)**	19.00	16.21	6.77	46.54	9.59	1.85	**25.06**	**15.65**	**4.90**	**125.52**	**26.23**	**5.14**

**Carbohydrate - glucose (mcg)**	71.35	68.93	29.85	152.54	32.19	6.20	**112.86**	**71.81**	**38.10**	**662.59**	**122.82**	**24.09**
**Potassium (mg)**	2667.11	2587.55	1366.62	4640.72	805.13	154.95	**3645.65**	**3037.37**	**1155.01**	**12996.94**	**2481.81**	**486.72**
**Energy_kcal**	1421.12	1368.66	478.76	2536.29	514.90	99.09	**2335.85**	**1714.79**	**870.56**	**12727.57**	**2284.66**	**448.06**
**Energy_kj**	5984.48	5760.81	2020.33	10707.78	2168.77	417.38	**9520.75**	**7032.37**	**3664.38**	**53529.71**	**9676.59**	**1897.74**
**Carbohydrate - lactose (g)**	3.54	2.62	0.23	9.65	2.97	0.57	**6.23**	**3.82**	**0.96**	**45.08**	**8.68**	**1.70**
**Carbohydrate - maltose (g)**	1.46	1.05	0.06	6.30	1.37	0.26	**3.52**	**2.03**	**0.17**	**24.30**	**4.95**	**0.97**
**Magnesium (mg)**	233.13	248.76	104.81	376.93	72.15	13.89	**319.16**	**267.28**	**100.45**	**1139.36**	**222.12**	**43.56**
**Manganese (mg)**	2.72	2.82	1.12	4.37	0.97	0.19	**3.73**	**2.71**	**0.71**	**14.70**	**3.22**	**0.63**
**Sodium (mg)**	2179.25	1980.43	583.67	3557.61	891.52	171.57	**3034.50**	**2234.15**	**1106.84**	**13993.37**	**2521.82**	**494.57**
**Niacin (mg)**	22.25	22.91	7.36	65.05	10.31	1.98	**26.99**	**21.90**	**9.13**	**81.92**	**16.18**	**3.17**
**Phosphorus (mg)**	1075.01	1076.07	453.21	2312.87	416.84	80.22	**1454.64**	**1104.10**	**528.78**	**6624.44**	**1175.60**	**230.55**
**Protein (g)**	76.80	75.34	25.83	216.21	35.86	6.90	**96.63**	**76.21**	**38.75**	**363.30**	**65.57**	**12.86**
**Vitamin A - retinol (mcg)**	1243.68	1019.15	38.37	5250.17	1262.40	242.95	**1591.90**	**615.90**	**54.90**	**14070.95**	**2883.79**	**565.56**
**Vitamin A - retinol equivalents (mcg)**	1561.85	1430.84	272.41	5370.13	1225.61	235.87	**1989.79**	**1189.57**	**161.84**	**14829.73**	**2969.61**	**582.39**
**Vitamin B2 - riboflavin (mg)**	1.34	1.34	0.61	2.47	0.52	0.10	**1.681**	**1.102**	**0.605**	**8.31**	**1.536**	**0.301**
**Selenium (mcg)**	66.49	67.71	22.55	151.51	26.03	5.00	**75.297**	**53.864**	**29.947**	**263.07**	**48.280**	**9.469**
**Carbohydrate - starch (g)**	90.07	82.68	33.43	237.32	42.40	8.16	**137.379**	**105.191**	**13.841**	**800.47**	**144.798**	**28.397**
**Carbohydrate - sucrose (g)**	32.92	23.01	9.77	160.22	28.59	5.503	**72.45**	**33.13**	**12.13**	**605.67**	**119.55**	**23.45**

**Vitamin B1 - thiamin (mg)**	1.16	1.14	0.56	1.95	0.38	0.07	**93.59**	**1.30**	**0.47**	**2395.00**	**469.40**	**92.06**
**Nitrogen (g)**	12.44	12.17	4.14	34.78	5.77	1.11	**15.73**	**12.31**	**6.36**	**59.56**	**10.75**	**2.11**
**Carbohydrate - sugars (total) (g)**	81.08	68.63	31.33	273.17	47.98	9.23	**138.14**	**79.95**	**33.95**	**939.79**	**183.90**	**36.07**
**Vitamin B12 - cobalamin (mcg)**	6.82	6.61	1.45	19.61	4.14	0.80	**8.45**	**6.07**	**1.65**	**52.37**	**10.24**	**2.01**
**Vitamin B6 - pyridoxine (mg)**	1.58	1.55	0.77	3.71	0.58	0.11	**2.13**	**1.58**	**0.66**	**7.14**	**1.38**	**0.27**
**Vitamin C - ascorbic acid (mg)**	84.85	76.94	38.37	207.07	38.76	7.46	**136.56**	**67.67**	**18.41**	**1111.13**	**212.87**	**41.75**
**Vitamin D - ergocalciferol (mcg)**	2.15	1.89	0.81	4.52	1.01	0.20	**3.51**	**2.19**	**1.35**	**22.85**	**4.29**	**0.84**
**Vitamin E - alpha tocopherol equivalents (mg)**	7.81	7.84	3.15	15.78	3.02	0.58	**13.00**	**8.35**	**3.34**	**75.19**	**14.13**	**2.77**
**Zinc (mg)**	8.06	8.02	2.89	17.55	3.12	0.60	**10.73**	**9.02**	**3.75**	**40.46**	**7.33**	**1.44**
**Fat - total (g)**	47.90	44.01	12.57	86.31	20.49	3.94	**86.03**	**59.03**	**29.39**	**513.83**	**94.52**	**18.54**
**Monounsaturated fatty acids (MUFA - total) (g)**	17.88	16.02	4.64	30.35	7.41	1.43	**34.33**	**22.55**	**8.86**	**210.71**	**39.90**	**7.83**
**Polyunsaturated fatty acids (PUFA - total) (g)**	8.57	7.97	2.61	15.13	3.51	0.68	**13.13**	**9.37**	**3.60**	**68.10**	**12.89**	**2.53**
**Saturated fatty acids (SFA - total) (g)**	16.60	14.40	3.70	39.98	8.92	1.72	**30.59**	**22.51**	**8.57**	**186.60**	**33.68**	**6.61**

**Alcoholic beverages (g)**	11.64	3.22	0.00	125.50	25.39	4.89	**43.48**	**20.16**	**0.00**	**316.00**	**67.39**	**13.22**
**Cereals and cereal products (g)**	204.18	178.11	21.18	975.65	176.05	33.88	**327.75**	**195.89**	**0.56**	**2585.28**	**485.73**	**95.26**
**Eggs and egg dishes (g)**	16.26	21.50	3.50	50.00	12.47	2.40	**16.31**	**17.50**	**0.00**	**50.00**	**13.91**	**2.72**
**Fats and oils (g)**	7.06	6.61	0.00	17.42	4.96	0.96	**17.78**	**6.21**	**0.21**	**177.12**	**37.08**	**7.27**
**Fish & fish products (g)**	24.62	22.40	0.00	54.74	15.86	3.05	**30.38**	**25.66**	**4.20**	**101.00**	**21.97**	**4.31**
**Fruit (g)**	163.25	144.55	43.65	326.05	82.08	15.80	**206.06**	**119.33**	**17.50**	**1477.80**	**323.22**	**63.39**
**Meat and meat products (g)**	140.12	122.71	20.65	613.13	103.53	19.92	**178.36**	**142.58**	**50.61**	**533.97**	**111.54**	**21.87**
**Milk and milk products (g)**	96.651	75.180	0.000	386.22	92.975	17.8930	**106.27**	**80.13**	**4.06**	**423.20**	**103.68**	**20.33**
**Non-alcoholic beverages (g)**	**670.93**	**519.80**	**196.30**	**1791.10**	**398.91**	**76.77**	597.59	498.80	9.48	1752.20	460.31	90.27
**Nuts and seeds (g)**	7.73	3.43	0.00	30.00	9.32	1.79	**19.29**	**2.10**	**0.00**	**137.66**	**35.30**	**6.92**
**Potatoes (g)**	69.76	71.39	17.50	148.94	35.89	6.91	**114.06**	**83.51**	**0.00**	**392.69**	**96.94**	**19.01**
**Soups & sauces (g)**	**102.75**	**90.20**	**0.00**	**230.10**	**63.31**	**12.18**	91.64	67.90	0.00	377.90	88.32	17.32
**Sugars (g)**	28.10	12.23	0.00	133.96	40.02	7.70	**73.75**	**43.35**	**0.00**	**530.50**	**108.06**	**21.19**
**preserves and snacks (g)**	210.16	224.45	26.10	383.01	102.42	19.71	**242.14**	**197.35**	**20.64**	**747.81**	**171.58**	**33.65**
**Vegetables**	3.89	5.00	0.00	5.00	2.12	0.41	**4.81**	**5.00**	**0.00**	**5.00**	**0.98**	**0.19**
**DII**	**2.27**	**2.07**	**-3.37**	**6.73**	**2.31**	**0.44**	1.85	2.24	-3.08	6.52	2.50	0.49
**hs troponin I**	**4.57**	**3.90**	**0.80**	**18.70**	**4.16**	**0.80**	1.62	1.10	0.10	7.80	1.59	0.31
**Cholesterol**	5.43	5.30	3.00	10.10	1.57	0.30	**5.89**	**5.90**	**3.20**	**8.40**	**1.33**	**0.26**

**HDL**	**1.28**	**1.20**	**0.80**	**2.10**	**0.34**	**0.07**	1.20	1.20	0.80	1.80	0.26	0.05
**LDL**	3.35	2.90	1.00	7.70	1.38	0.27	**3.78**	**3.85**	**2.00**	**6.00**	**1.09**	**0.21**
**TG**	1.90	1.80	0.80	3.70	0.77	0.15	**2.16**	**2.20**	**0.80**	**4.60**	**0.86**	**0.17**
**TSH**	2.11	1.71	0.08	7.69	1.50	0.28	**2.68**	**2.51**	**0.75**	**5.74**	**1.22**	**0.24**
**fT4**	**13.48**	**13.42**	**9.48**	**18.57**	**2.06**	**0.40**	12.09	12.06	9.32	14.68	1.46	0.29
**fT3**	4.01	4.21	2.64	5.51	0.78	0.15	**4.44**	**4.34**	**3.43**	**5.59**	**0.60**	**0.12**
**Insulin**	17.07	14.20	4.30	31.20	7.60	1.46	**17.75**	**16.40**	**4.50**	**56.30**	**10.17**	**1.99**
**HOMA-IR**	18.30	7.10	1.70	113.40	28.60	5.50	**37.91**	**34.58**	**1.40**	**127.21**	**30.06**	**5.90**
**Weight**	**128.04**	**127.20**	**95.30**	**177.50**	**18.15**	**3.49**	125.15	121.00	95.90	183.60	20.66	4.05
**SMM**	37.09	34.90	23.80	53.10	7.68	1.48	37.47	35.10	26.90	56.20	8.72	1.71
**BFM**	**62.32**	**64.20**	**40.50**	**85.20**	**10.98**	**2.11**	59.18	58.40	40.40	90.30	12.05	2.36
**PBF%**	47.84	50.00	34.10	56.30	6.07	1.17	**48.05**	**49.85**	**30.30**	**64.50**	**7.22**	**1.42**
**BMI**	**46.63**	**46.10**	**36.40**	**57.50**	**5.81**	**1.12**	42.45	42.25	35.40	54.80	4.98	0.98
**BMR**	1789.70	1704.00	1299.00	2363.00	276.67	53.25	**1794.96**	**1701.00**	**1418.00**	**2462.00**	**310.49**	**60.89**
**WAIST-TO-HIP-RATIO**	1.03	1.04	0.70	1.26	0.12	0.02	**1.08**	**1.07**	**0.92**	**1.27**	**0.09**	**0.02**
**VFL**	19.63	20.00	16.00	20.00	1.08	0.21	**19.92**	**20.00**	**19.00**	**20.00**	**0.27**	**0.05**
**M1**	**630.04**	**762.00**	**197.00**	**912.00**	**259.53**	**49.95**	444.39	333.50	186.00	883.00	239.46	46.96
**M2**	276.78	219.00	84.00	561.00	160.31	30.85	**358.65**	**370.50**	**113.00**	**575.00**	**142.85**	**28.02**
**M3**	33.04	12.00	1.00	113.00	36.81	7.084	**71.35**	**72.50**	**2.00**	**140.00**	**39.74**	**7.79**
**M4**	59.37	13.00	0.00	271.0	80.15	15.43	**125.62**	**108.00**	**0.00**	**302.00**	**97.99**	**19.22**
**NDI**	1.52	1.27	1.09	2.44	0.45	0.09	**1.88**	**1.95**	**1.12**	**2.55**	**0.45**	**0.09**

**MN**	**10.91**	**10.00**	**3.00**	**24.00**	**5.62**	**1.08**	7.02	6.25	1.50	14.50	3.65	0.72
**NB**	**5.70**	**3.00**	**0.00**	**21.00**	**6.20**	**1.19**	5.50	4.50	1.00	14.00	3.82	0.75
**NPB**	**5.96**	**5.00**	**0.50**	**16.00**	**4.25**	**0.82**	5.50	3.50	1.00	17.00	4.44	0.87
**APOPTOSIS**	**9.56**	**8.00**	**0.00**	**42.00**	**8.87**	**1.71**	6.19	5.00	2.00	18.00	3.84	0.75
**NECROSIS**	**3.82**	**0.00**	**0.00**	**33.00**	**8.61**	**1.66**	1.73	0.00	0.00	29.00	6.00	1.18
**Net Fpg, TI, %**	**6.83**	**4.22**	**0.00**	**31.59**	**7.77**	**1.49**	5.71	4.59	0.23	13.50	4.08	0.80
**TI, %**	**11.16**	**10.53**	**4.55**	**18.27**	**4.05**	**0.78**	9.83	9.24	4.50	17.88	3.92	0.77
**Age, years**	**60.81**	**61.00**	**51.00**	**68.00**	**4.49**	**0.86**	41.11	41.53	26.35	51.41	7.26	1.42

Table S8. Spearman's correlation coefficient between different nutrient intakes derived by food frequency questionnaire and anthropometric, biochemical or DNA parameters

Variable	Mean	Median	Min	Max	SD	SE	Spearman correlation
Alpha carotene (mcg)	141.16	105.85	4.62	994.71	161.59	22.20	HDL(0.30), HOMA-IR(0.32), apoptosis(-0.29), Energy kcal(-0.28), Net Fpg(-0.28)
Alcohol (g)	4.30	0.79	0.00	43.75	9.34	1.283	LDL(-0.28)
Beta carotene (mcg)	1849.03	1572.68	301.25	5381.19	1229.58	168.90	HDL(0.35), Net Fpg(-0.36)
Calcium (mg)	648.05	514.61	180.57	3734.16	519.71	71.39	HOMA-IR(0.32)
Carotene - total (carotene equivalents) (mcg)	2131.80	1820.92	359.58	5945.25	1379.89	189.54	HDL(0.28), Net Fpg(-0.34)
Carbohydrate - total (g)	229.08	179.68	54.23	1771.66	240.14	32.99	Net Fpg(-0.30)
Cholesterol (mg)	324.01	247.48	118.47	2284.20	304.06	41.77	
Chloride (mg)	3754.42	3088.07	978.96	18279.11	2559.73	351.61	
Copper (mg)	1.69	1.31	0.39	11.89	1.67	0.23	NPB(0.34)
Englyst Fibre - Non Starch Polysaccharides (NSP) (g)	807.44	14.04	3.81	42003.00	5767.47	792.22	
Iron (mg)	11.22	9.51	3.71	54.90	7.36	1.01	
Total folate (mcg)	247.68	209.47	73.20	726.66	117.49	16.14	
Carbohydrate - fructose (g)	22.07	16.08	4.82	110.14	19.27	2.65	NPB(0.29)
Carbohydrate - galactose (g)	0.65	0.27	0.00	4.41	0.89	0.12	
Carbohydrate - glucose (g)	21.97	15.89	4.90	125.52	19.65	2.70	HOMA-IR(0.3), Net Fpg(-0.32), NB(-0.37), NPB(0.27)

Carbohydrate - glucose (mcg)	91.71	70.40	29.85	662.59	90.61	12.45	
Potassium (mg)	3147.15	2662.94	1155.01	12996.94	1878.64	258.05	
Energy_kcal	1869.86	1511.38	478.76	12727.57	1689.72	232.10	Age (-0.30)
Energy_kj	7719.25	6140.72	2020.33	53529.71	7110.17	976.66	
Carbohydrate - lactose (g)	4.86	3.14	0.23	45.08	6.52	0.90	HOMA-IR(0.28), Net Fpg(-0.27), mean comet(-0.28)
Carbohydrate - maltose (g)	2.47	1.27	0.06	24.30	3.72	0.51	Hs troponin I(-0.28), mean comet(-0.39), age(-0.35)
Magnesium (mg)	275.33	258.45	100.45	1139.36	167.95	23.07	NPB(0.28)
Manganese (mg)	3.21	2.77	0.71	14.70	2.39	0.33	Net Fpg(-0.32)
Sodium (mg)	2598.81	2202.57	583.67	13993.37	1908.19	262.11	
Niacin (mg)	24.58	22.24	7.36	81.92	13.59	1.87	
Phosphorus (mg)	1261.24	1079.61	453.21	6624.44	887.71	121.94	
Protein (g)	86.53	75.40	25.83	363.30	53.01	7.28	
Vitamin A - retinol (mcg)	1414.50	986.15	38.37	14070.95	2196.79	301.75	SMM(0.29), PBF%(-0.29), BMR(0.31)
Vitamin A - retinol equivalents (mcg)	1771.79	1381.10	161.84	14829.73	2244.42	308.29	SMM(0.27), BMR(0.28)
Vitamin B2 - riboflavin (mg)	1.51	1.16	0.61	8.31	1.14	0.16	
Selenium (mcg)	70.81	66.82	22.55	263.07	38.46	5.28	
Carbohydrate - starch (g)	113.28	91.10	13.84	800.47	107.47	14.76	Net Fpg(-0.31)
Carbohydrate - sucrose (g)	52.31	29.11	9.77	605.67	87.62	12.04	mean comet(-0.35), age(-0.30)
Vitamin B1 - thiamin (mg)	46.50	1.16	0.47	2395.00	328.80	45.16	

Nitrogen (g)	14.06	12.17	4.14	59.56	8.66	1.19	
Carbohydrate - sugars (total) (g)	109.07	73.76	31.33	939.79	135.06	18.55	mean comet(-0.32)
Vitamin B12 - cobalamin (mcg)	7.62	6.22	1.45	52.37	7.72	1.06	fT3(-0.29), SMM(0.28), BMR(0.29), MN(0.28)
Vitamin B6 - pyridoxine (mg)	1.85	1.57	0.66	7.14	1.07	0.15	BFM(0.29)
Vitamin C - ascorbic acid (mg)	110.21	69.94	18.41	1111.13	152.37	20.93	M3(-0.32), M4(-0.33), NDI(-0.30), Net Fpg(-0.28)
Vitamin D - ergocalciferol (mcg)	2.82	2.02	0.81	22.85	3.13	0.43	SMM(0.28), BMR(0.28)
Vitamin E - alpha tocopherol equivalents (mg)	10.36	8.02	3.15	75.19	10.37	1.42	W-H-ratio(0.27)
Zinc (mg)	9.37	8.30	2.89	40.46	5.71	0.78	
Fat - total (g)	66.61	51.17	12.57	513.83	69.83	9.59	Age(-0.29)
Monounsaturated fatty acids (MUFA - total) (g)	25.95	19.64	4.64	210.71	29.36	4.03	Age(-0.29)
Polyunsaturated fatty acids (PUFA - total) (g)	10.807	8.652	2.599	68.10	9.559	1.31	
Saturated fatty acids (SFA - total) (g)	23.46	18.02	3.70	186.60	25.20	3.46	Hs troponin I(-0.31), age(-0.37)
